# Sources of Type I Interferons in Infectious Immunity: Plasmacytoid Dendritic Cells Not Always in the Driver's Seat

**DOI:** 10.3389/fimmu.2019.00778

**Published:** 2019-04-12

**Authors:** Shafaqat Ali, Ritu Mann-Nüttel, Anja Schulze, Lisa Richter, Judith Alferink, Stefanie Scheu

**Affiliations:** ^1^Institute of Medical Microbiology and Hospital Hygiene, University of Düsseldorf, Düsseldorf, Germany; ^2^Cluster of Excellence EXC 1003, Cells in Motion, Münster, Germany; ^3^Department of Psychiatry, University of Münster, Münster, Germany

**Keywords:** type I interferon, plasmacytoid dendritic cells, interferon producing cells, infection, pathogen, virus, immunopathology, immune activation

## Abstract

Type I Interferons (IFNs) are hallmark cytokines produced in immune responses to all classes of pathogens. Type I IFNs can influence dendritic cell (DC) activation, maturation, migration, and survival, but also directly enhance natural killer (NK) and T/B cell activity, thus orchestrating various innate and adaptive immune effector functions. Therefore, type I IFNs have long been considered essential in the host defense against virus infections. More recently, it has become clear that depending on the type of virus and the course of infection, production of type I IFN can also lead to immunopathology or immunosuppression. Similarly, in bacterial infections type I IFN production is often associated with detrimental effects for the host. Although most cells in the body are thought to be able to produce type I IFN, plasmacytoid DCs (pDCs) have been termed the natural “IFN producing cells” due to their unique molecular adaptations to nucleic acid sensing and ability to produce high amounts of type I IFN. Findings from mouse reporter strains and depletion experiments in *in vivo* infection models have brought new insights and established that the role of pDCs in type I IFN production *in vivo* is less important than assumed. Production of type I IFN, especially the early synthesized IFNβ, is rather realized by a variety of cell types and cannot be mainly attributed to pDCs. Indeed, the cell populations responsible for type I IFN production vary with the type of pathogen, its tissue tropism, and the route of infection. In this review, we summarize recent findings from *in vivo* models on the cellular source of type I IFN in different infectious settings, ranging from virus, bacteria, and fungi to eukaryotic parasites. The implications from these findings for the development of new vaccination and therapeutic designs targeting the respectively defined cell types are discussed.

## Introduction

The cytokine family of type I IFNs fulfills key functions in anti-viral immunity but is also produced in the immune responses to other classes of pathogens covering viruses, bacteria, parasites, and fungi (1). Additionally, these cytokines are functionally involved in the pathogenesis of inflammatory autoimmune diseases (2).

Together with IFNβ, type I IFNs comprise multiple IFNα subtypes (11 in mice and 13 in humans), IFNε, IFNκ, and IFNω in most mammals. In addition, IFNδ, IFNζ (limitin), and IFNτ have been detected exclusively in pigs, mice, and ruminants, respectively (3–6). Type I IFNs are encoded by intronless genes clustered in mice on chromosome 4 and in humans on chromosome 9 (3–6). Induction of type I IFN expression is facilitated after activation of a diverse set of pathogen sensing pattern recognition receptor (PRR) pathways by binding of IFN regulatory factors (IRFs) and NF-κB to acute response elements in the promoters of type I IFN gene loci (7). All type I IFNs bind to a common heterodimeric IFNα receptor (IFNAR), which is composed of the IFNAR1 and IFNAR2 subunits and is expressed by virtually all nucleated cells of the body. Following IFNAR engagement by its ligands, canonical type I IFN signaling activates the Janus kinase (JAK)-signal transducer and activator of transcription (STAT) pathway, leading to transcription of IFN-stimulated genes (ISGs) (5, 8). ISG-encoded proteins mediate induction of cell-intrinsic antimicrobial states in infected and neighboring cells that limit the spread of infectious agents, particularly viral pathogens. Additionally, ISGs influence innate and adaptive immune responses by promoting antigen presentation and NK cell functions, modulating inflammatory cytokine production, and activating high-affinity antigen-specific T and B cell responses and immunological memory (9). Type I IFN production, however, can also have deleterious roles in chronic viral and bacterial infections, and can lead to immunopathologies such as inflammatory disorders and autoimmunity (1, 2, 10, 11).

IFNβ was originally defined as the antiviral factor produced by fibroblasts after viral infections (12) and has been thought to be produced by virtually all cells of the body. Later pDCs specialized in the rapid secretion of high amounts of type I IFN have been termed the natural “IFN producing cells” (IPCs). Recent findings, however, indicate that production of type I IFN, especially the early synthesized IFNβ, in anti-infectious immune responses can occur independently of pDCs and that the cell type responsible for type I IFN production rather depends on the specific infectious setting. In this review we summarize the recent findings on the identity and function of type I IFN producing cells in infection by focusing on insights gained from *in vivo* mouse models covering type I IFN reporter mice and models of cell type specific ablation.

### Pathways of Type I IFN Activation in Different Cell Types

To devise novel anti-infectious treatment regimens targeting a specific cellular subtype, it is crucial to know the identity of the cells responsible for the production of type I IFN in the course of an infection. Early on, pDCs were considered primary producers of IFNα during virus infections (13, 14). For human pDCs it has been reported that IFNα/β transcripts account for an astounding 50% of all mRNAs in the cell after viral activation (15). More than 40 years ago, pDCs were first described in humans as natural IPCs that activate NK cells upon exposure to viruses (16, 17). The murine equivalent was described in 2001 as type I IFN producing cells with plasmacytoid morphology (18–20). These cells detect RNA and DNA viruses through two endosomal sensors, TLR7 and TLR9, respectively, which induce secretion of type I IFN through the MyD88-IRF7 signaling pathway (21–24). Specifically, TLR7/9-ligand interactions in early endosomes result in type I IFN production while ligand recognition in late endosomes or lysosomes rather leads to inflammatory cytokine production and pDC maturation (25, 26). At least in the mouse, TLR7 and 9 are also expressed by monocytes, conventional DCs (cDCs), and B cells (27, 28). Therefore, the contribution of those cell types to type I IFN production triggered via the TLR7/9-MyD88-IRF7 pathway has to be considered. B cells, for instance, have recently been shown to produce type I IFN *in vivo* after optimized stimulation conditions using the TLR9 ligand CpG-A (29). A specific feature of pDCs is that they can produce type I IFN independently of IFNAR mediated feedback signaling (30). However, they do respond to type I IFN by generating an autocrine circuit through IFNAR, which augments type I IFN secretion and induces their activation and migration (31, 32).

In humans, pDCs, monocytes, and other myeloid cells also produce type I IFN after stimulation of the TLR8-MyD88-IRF7 pathway by viral single-stranded RNA (ssRNA) (33, 34). The mouse TLR8 was initially considered non-functional (33, 34). More recently it has been shown that mouse TLR8 can be stimulated by a combination of oligodeoxynucleotides (ODNs) and human TLR8 ligands. Further, mouse pDCs produce type I IFN after stimulation with vaccinia virus (VV) in a TLR8 dependent way (35, 36). Two additional TLRs, TLR3 and 4, are able to induce type I IFN expression independently of the MyD88 pathway via recruiting the TIR domain-containing adaptor protein inducing interferon beta (TRIF; also known as TIR domain-containing adapter molecule 1, TICAM-1). This activates the transcription factor IRF3 thus initiating type I IFN, in particular IFNβ expression (37, 38). TLR3 is absent in mouse pDCs but highly expressed in endosomes of murine CD8α^+^ and CD103^+^ and human CD141^+^ cDCs of the DC1 subtype that are efficient in cross-presenting (39, 40). It recognizes double-stranded RNA (dsRNA) as viral replication intermediates as well as ssRNA containing stem loops (41). In addition to DCs, TLR3 activation can lead to type I IFN expression in macrophages, fibroblasts, and epithelial cells (42). While TLR3 exclusively signals via the TRIF pathway, TLR4 utilizes MyD88 as well as TRIF signaling routes after recognizing its cognate ligand bacterial lipopolysaccharide (LPS). Analogous to TLR3 activation, LPS binding to TLR4 induces type I IFN expression via TRIF-IRF3 (43). The majority of hematopoietic cells of the myeloid and lymphoid lineage, with the exception of human pDCs, and few other cell types such as pancreatic β-cells express TLR4 (44).

In contrast to pDCs, cDCs, and macrophages mainly produce type I IFN in response to virus challenge by utilizing retinoic acid-inducible gene I (RIG-I)-like helicases (RLHs) (43, 45–47). RLHs, including RIG-I and melanoma differentiation-associated gene 5 (MDA5), are cytoplasmic dsRNA receptors that transmit their signal through the mitochondrial antiviral-signaling protein, virus-induced signaling adapter (MAVS, aka *IFNb* promoter stimulator (IPS)-1 or Cardif). This activates IRF3 and IRF7 to induce the transcription of type I IFN and other antiviral genes (48–50).

Finally, soluble sensors in the cytoplasm detect dsDNA in a sequence-independent manner, exhibit a broad expression spectrum including pDCs, cDCs, macrophages, and mouse embryonic fibroblasts (MEFs), and activate signaling pathways leading to type I IFN expression (47, 51). These sensors include the cyclic GMP-AMP synthase (cGAS)/STING pathway, the RNA polymerase III/RIG-I/MAVS pathway, DNA-dependent activator of IRFs (DAI), IFNγ-inducible protein 16 (IFI16), and the DDX family (47, 51–58).

### Mouse Models and *in vivo* Experimental Strategies for the Definition of the Cellular Source of Type I IFNs in Infection

**Figure 1:**
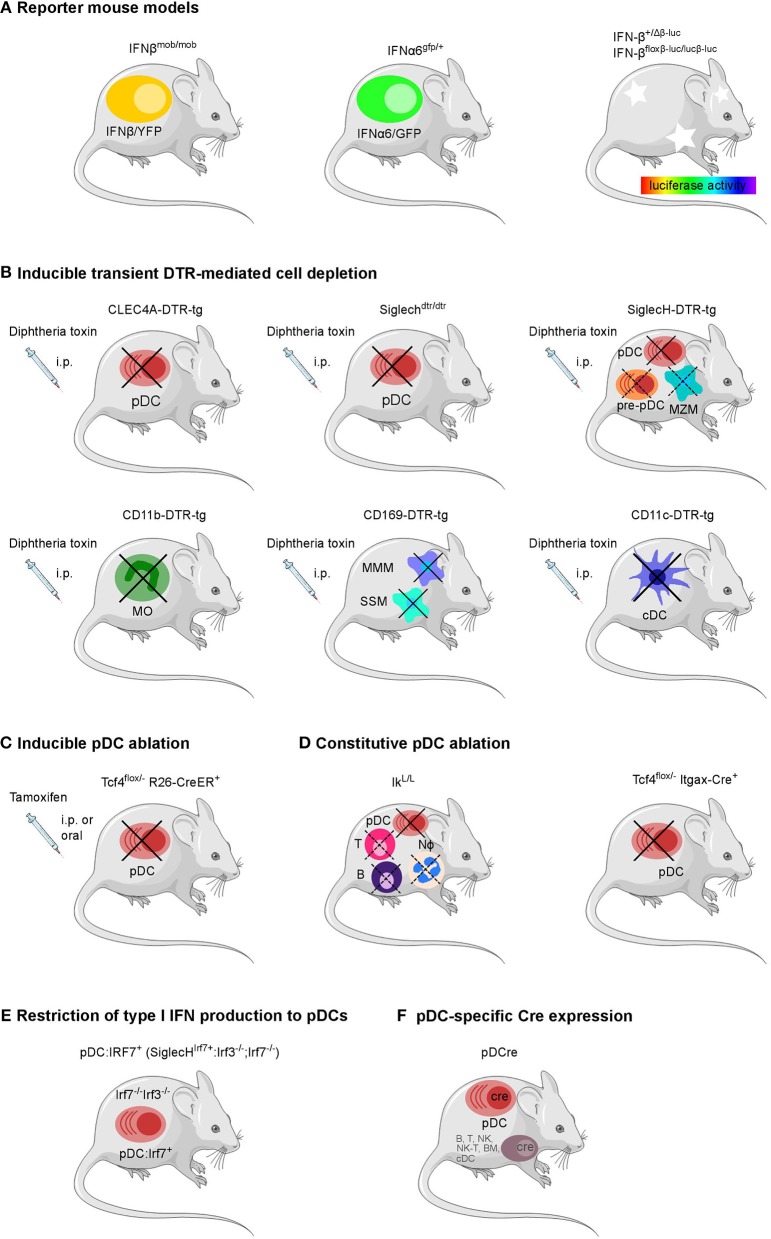
Overview of genetically modified mouse models available to define the cellular source and impact of type I IFNs. **(A)** Reporter mouse models for the detection of type I IFN producing cells, **(B)** mouse strains for the transient DTR-mediated cell depletion and **(C)** inducible and **(D)** constitutive ablation of pDCs, **(E)** pDC-specific Cre expression, and **(F)** a mouse line with a restriction of the type I IFN production to pDCs have been employed in various infection settings *in vivo*.Each model system harbors specific advantages and caveats as further described in Table 1. B, B cell; BM, bone marrow cell; cDC, conventional dendritic cell; MMM, marginal metallophilic macrophage; MZM, marginal zone macrophage; MO, monocyte; NK, natural killer cell; Nφ, neutrophil; SSM, subcapsular sinus macrophage; T, T cell; pDC, plasmacytoid dendritic cell. The figure was created using Servier Medical Art according to Creative Commons Attribution 3.0 Unported License (https://creativecommons.org/licenses/by/3.0/). Changes were made to the original cartoons.

Several models of cytokine reporter mice have been developed for the detection of type I IFN production *in vivo*, as intracellular staining is not sensitive in most cases (Table 1 and Figure 1). Earlier published IFNβ knock-out mouse lines already contained reporter elements to detect *Ifnb* promoter-driven gene transcription. For example, coding sequences for the mouse immunoglobulin λ2 chain, a green fluorescent protein (GFP), or the human CD2 had been inserted immediately downstream of the *Ifnb* promoter to visualize IFNβ expression on a cellular level (73–75). However, the reporter features in these mouse strains have not been used *in vivo* so far.

**Table 1:** Genetically modified mouse models to visualize or define the function of type I IFN producing cells.

**Function**	**Name(s)**	**Genetic modification**	**References**
IFNα6-GFP reporter mouse	Ifna6^gfp/+^	Knock-in of a GFP reporter gene into the *Ifna6* locus behind the *Ifna6* promoter; endogenous IFNα6 expression retained in homozygous reporter mouse	(46)
IFNβ-YFP reporter mouse	IFNβ^mob/mob^ (B6.129-Ifnb1tm1Lky/J)	Knock-in of an IRES-driven YFP reporter cassette behind the Stop codon of the *Ifnb1* gene; endogenous IFNβ expression retained in homozygous reporter mouse	(59)
IFNβ-luciferase reporter and conditional IFNβ knock-out	IFN-β^+/Δβ−*luc*^ and IFN-β^floxβ−*luc*/*floxβ*−*luc*^	Knock-in of firefly luciferase reporter gene into the *Ifnb1* locus behind the *Ifnb1* promoter; endogenous IFNβ expression retained in homozygous reporter mouse; IFNβ coding sequence has been flanked by *loxP* sites.	(60)
Constitutive pDC ablation (*caveat*: T and B cells, and neutrophils affected)	Ik^L/L^	Knock-in of the βgal coding sequence in-frame into exon-2 of the *Ikaros* gene resulting in a hypomorphic mutation	(61, 62)
Constitutive pDC ablation	Tcf4^flox/−^ Itgax-Cre^+^	Floxed *Tcf4* gene crossed to DC specific Itgax-CRE mice	(63, 64)
Inducible pDC ablation	Tcf4^flox/−^*R26*-CreER^+^	Floxed *Tcf4* gene crossed to tamoxifen-inducible *R26*-CreER mice	(64)
Inducible transient pDC depletion after diphtheria toxin (DT) administration	CLEC4A-DTR-tg (BDCA2-DTR tg)	Transgene containing a 5 kb fragment upstream of the ATG of the human *CLEC4A* (BDCA-2) gene followed by the diphtheria toxin receptor (DTR) cDNA	(65)
Inducible transient pDC depletion after DT administration	Siglech^dtr/dtr^	Knock-in of an IRES-driven cDNA encoding the human DTR fused to the enhanced green fluorescent protein (EGFP) into the 3' untranslated region of the *Siglech* gene	(66)
Inducible transient pDC depletion after DT administration (*caveat*: MZM and pre-pDC affected)	SiglecH-DTR-tg	BAC transgene, modified BAC encoding the complete *Siglech* gene locus (RPA24-163A12), bicistronic cassette containing cDNAs for human DTR and EGFP inserted into *Siglech* exon I, after the second triplet of the open reading frame	(67)
pDC-specific Cre expression (*caveat*: Cre mediated recombination detected in a minor fraction of SiglecH^−^ B-, T-, NK-, and NK-T cells and splenic cDC and CD11c^int^ BM cells)	pDCre	BAC transgene, modified BAC encoding the complete *Siglech* gene locus (RP24-396N13), bicistronic cassette containing cDNAs for Cre and mCherry inserted into *Siglech* exon I, after the ATG	(68)
Constitutive restriction of type I IFN production to pDCs and tamoxifen inducible pDC specific Cre expression	pDC:IRF7^+^ (Siglech^Irf7/+^; Irf3^−/−^; Irf7^−/−^)	Knock-in of bicistronic cassette containing the *irf7* gene locus stretching (protein coding exons) and the cDNA for Cre fused to the mutated ligand binding domain of the human estrogen receptor (ERT2), backcrossed Irf3^−/−^; Irf7^−/−^ double knockout mice	(69)
Inducible transient cDC depletion after DT administration	CD11c-DTR-tg	Transgene containing the murine CD11c promoter followed by a cDNA coding for a DTR-EGFP fusion protein	(70)
Inducible transient monocyte depletion after DT administration	CD11b-DTR-tg	Transgene containing the human CD11b promoter followed by a cDNA coding for a DTR-EGFP fusion protein	(71)
Inducible transient monocyte depletion of marginal metallophilic macrophages in the spleen and subcapsular sinus macrophages in the lymph nodes after DT administration	CD169-DTR-tg	Knock-in of the cDNA for the human DTR into the endogenous gene locus, behind the promoter for CD169.	(72)

More recently, a mouse line expressing GFP under the control of the *Ifna6* promoter (Ifna6^gfp/+^) recapitulates the expression of various IFNα genes and has been employed to define the cellular source of IFNα in virus infection models (32, 46, 76). Also, for IFNβ a fluorescence reporter-knock-in mouse model (IFNβ^mob/mob^) has been generated. Here, yellow fluorescent protein (YFP) is expressed from a bicistronic mRNA linked by an internal ribosomal entry site (IRES) to the endogenous IFNβ mRNA (59). Ifna6^gfp/+^ as well as IFNβ^mob/mob^ reporter mice have each been shown to report for the majority of type I IFNs. However, *in vitro* analyses on BM-derived DCs from the double reporter mouse line generated by intercrossing the Ifna6^gfp/+^ and IFNβ^mob/mob^ reporter strains revealed that specific type I IFN subtypes can be produced by distinct cell subpopulations (77).

In an alternative reporter mouse system, a firefly luciferase reporter gene has been placed under the control of the *Ifnb* promoter (IFN-β^+/Δβ−luc^). Rather than IFNβ expression on a single cell level, this model detects *in vivo* kinetics of IFNβ expression in the mouse paralleling the spread of pathogens through the organism under infectious conditions (60). Additionally, in this mouse line the IFNβ coding sequence is flanked by *loxP* sites (IFN-β^floxβ−luc/floxβ−luc^) providing the possibility to characterize the impact of IFNβ production by a given cell type on the pathophysiology of various infections via tissue- or cell-specific Cre-mediated deletion of IFNβ (60).

### Methods and Models for Assessing the Impact of Type I IFN Producing Cell Populations *in vivo*

Several experimental strategies have been developed to determine the *in vivo* contribution of a specific cell type to the type I IFN response during infections (78). Initially, antibody mediated depletion has been utilized frequently to ablate pDCs and monocytes (79). Antibodies against Ly6G/C (also known as Gr1) and CD317 (also known as BST-2) have been used to deplete pDCs *in vivo* and *in vitro* (18, 79–87). However, these antibodies generally target multiple cell types in addition to pDCs: The antibody RB6-8C5 directed against Ly6G/C reacts strongly with neutrophil-specific Ly6G antigen, but cross-reacts also with the Ly6C Ag (88) expressed on pDCs as well as on monocytes/macrophages, activated T cells, NK cells, plasma cells, and endothelial cells (89–92). Likewise, CD317 is recognized by the three different antibody clones 120G8.04, JF05-1C2.4.1 (also known as PDCA-1), and eBio927, and is expressed in naïve mice by pDCs, but also plasma cells. Following stimulation with type I IFNs and IFNγ CD317 is upregulated, additionally, on several other myeloid and lymphoid cells (79, 93). Finally, *in vivo* treatment with clodronate-containing liposomes depletes phagocytes in mice, but also disturbs the microarchitecture of secondary lymphoid organs (94, 95).

In the past years, several genetically modified mouse lines with a constitutive or inducible lack of specific cell types attributed to produce type I IFN have become available (Table 1 and Figure 1) (78). For pDCs, already several mouse models exist for constitutive or inducible ablation. Mice carrying a hypomorphic mutation at the *Ikaros* locus express low levels of the transcription factor Ikaros (Ik^L/L^) and therefore lack peripheral pDCs, but no other DC subsets (61). When using this line as a “pDC-less” model, one has to take into account that other hematopoietic lineages including T and B cells and neutrophils are also affected by the Ik^L/L^ mutation, and that Ik^L/L^ mice start to develop thymic lymphomas by 10 weeks of age (62, 96, 97). Constitutive deletion of E2-2, the basic helix-loop-helix transcription factor, also known as TCF4, that controls development and maintenance of pDCs, results in perinatal lethality in mice (98). To overcome this lethality and to specifically ablate the pDC lineage, mice harboring a constitutively deleted and a floxed *Tfc4* allele (Tcf4^flox/−^) have been crossed to Itgax-Cre (CD11c-Cre) or Rosa26-CreER mice in which Cre is expressed in DCs or can be induced ubiquitously after tamoxifen administration, respectively (63, 64, 99, 100). Another strategy uses Diphtheria toxin receptor (DTR)-mediated conditional and targeted cell depletion. CLEC4A-DTR-tg mice express DTR under the human pDC specific promotor of the C-type lectin domain family 4 member A (CLEC4A; also known as blood dendritic cell antigen 2, BDCA2). Administration of diphtheria toxin (DT) in these CLEC4A-DTR-tg mice results in transient but specific depletion of pDCs (65). In an alternative approach, a cDNA encoding the human DTR fused to the enhanced green fluorescent protein (EGFP) and preceded by an IRES was inserted into the 3′ untranslated region of the *Siglech* gene. This Siglech^dtr/dtr^ mouse model allows specific elimination of pDCs *in vivo* via injection of DT (66). An analogous mouse line termed SiglecH-DTR-tg was generated using bacterial artificial chromosome (BAC) transgenic technology (67). SiglecH represents a sialic acid–binding Ig-like lectin that exerts immunomodulatory roles in antiviral immune responses. In SiglecH^eGFP/+^mice, heterozygous for the reporter gene, it was shown that in addition to pDCs, SiglecH was expressed in specialized macrophage subsets, such as marginal zone macrophages (MZM), lymph node medullary macrophages, and microglia. SiglecH was also found in immediate precursors of pDCs (pre-pDCs) in the BM, which have the plasticity to differentiate into pDCs and cDCs (67, 101). Despite of SiglecH expression on above described other cell types Loschko et al. showed pDC specific antigen delivery in mice by using SiglecH as a target structure (102) suggesting the usability of SiglecH as a lead molecule for the generation of pDC specific transgenic animals. A side by side comparison showed a higher susceptibility to *Listeria monocytogenes* infection in DT-treated SiglecH-DTR-tg vs. CLEC4A-DTR-tg mice. This finding was attributed to the additional lack of MZM in SiglecH-DTR-tg mice after DT treatment which was not observed in CLEC4A-DTR-tg mice (67).

With the aim to specifically express the Cre recombinase in pDCs a BAC-tg “pDCre” mouse line was generated which expresses Cre under the control of the *Siglech* promoter (68). By crossing these mice with a reporter mouse line that indicates Cre activity via red fluorescent protein (RFP) expression the authors found ~30% of SiglecH^+^ pDCs terminally labeled with RFP. Additionally, RFP expression was observed in a minor fraction of SiglecH^−^ B-, T-, NK-, and NK-T cells and splenic cDCs and CD11c^int^ BM cells suggesting that a small fraction of early lymphoid progenitors actively transcribes the SiglecH locus. Thus, the broader expression pattern of SiglecH should be considered when using SiglecH-DTR-tg mice to evaluate pDC functions *in vivo*.

Recently, a novel mouse model has been described in which type I IFN production is restricted to pDCs. In this knock-in model Irf7 expression is driven by the *Siglech* promoter (Siglech^Irf7/+^). The Siglech^Irf7/+^ mice were then backcrossed onto Irf3^−/−^/Irf7^−/−^ double knock-out mice which are deficient in type I IFN production. This yielded animals (referred to as “pDC:Irf7^+^” mice) in which IRF7 signaling required for type I IFN expression is functional exclusively in pDCs (69). Additionally, in these mice an IRES site followed by coding sequences for Cre fused to the mutated ligand binding domain of the human estrogen receptor (ERT2) (103) was inserted behind the *Irf7* gene into the *Siglech* gene locus (69). Therefore, this mouse line can potentially be used in the future for tamoxifen inducible pDC specific Cre expression and thus pDC specific gene deletion when crossed to the respective floxed mouse lines.

Also, for other cell types than pDCs, the DTR-mediated depletion approach has been employed. In recent years, mice expressing the DTR under the control of the CD11b-, CD11c-, and CD169-promoters have been generated and successfully used for depletion of monocytes, cDCs, and CD169^+^ macrophage subpopulations such as MZMs and subcapsular sinus macrophages in the spleen and lymph nodes (70–72, 104–109).

In the following sections we will discuss approaches designed to define the type I IFN producing cell types in infection using the *in vivo* mouse models described above.

## Viral Infections

In this chapter we will focus on more recent findings from *in vivo* models aimed at visualizing IFNα/β producing cell types and defining their contribution to the overall type I IFN production and their impact on the course of viral infections (Table 2). For a more generalized overview of the cellular sources of type I IFN in viral infections we kindly refer to an expert review by Swiecki et al. (127).

**Table 2:** Cellular sources of type I IFN production in viral infections *in vivo*.

**Virus**	**Type I IFN producing cells**	**Model systems and assay methods**	**Observations in the absence of cell type**
Murine cytomegalovirus (MCMV)	Splenic stroma cells	RT-PCR from *ex vivo* purified splenic stroma cells 8 h p.i. (110)	n.d.
	pDCs	FACS and immunohistochemistry of the spleen of IFNβ^mob/mob^ reporter mice 12h p.i. (59, 111), ELISA from SN of *ex vivo* sorted Ly6G/C^+^ DCs 36h p.i. (112)	n.d.
		pDC depletion by anti-CD317 or anti-Ly6G/C (18, 80, 81, 113) or in CLEC4A-DTR-tg mice (65), pDC-deficient Ikaros^L/L^ mice (113)	↓↓ IFNα serum levels until 36 h p.i.; ↑ Viral burden in the absence of pDCs only at low viral inoculation
	non-pDCs	pDC depletion by anti-Ly6G/C (113) or in CLEC4A-DTR-tg mice (65), pDC-deficient Ikaros^L/L^ mice (113)	= IFNα serum levels starting 42 h p.i.
Herpes simplex virus-1 (HSV-1)	pDCs	Subcutaneous infection, pDC depletion by anti-CD317 or anti-Ly6G/C (82)	↑ viral titers in the draining LNs day 7 p.i.; type I IFN levels not measured
		i.v. infection, pDC depletion in Siglech^dtr/dtr^ (66) or CLEC4A-DTR-tg mice (65, 114)	↑ Viral titers day 6 p.i. ↓ IFNα serum levels 6 h p.i.
	non-pDCs	Subcutaneous infection, pDC depletion in CLEC4A-DTR-tg mice (65, 114)	No viral titers measurable in the draining LNs day 7 p.i. in depleted or control mice, comparable CTL activation; type I IFN levels not measured
		i.v. infection, TLR3^−/−^ mice (114)	↓ IFNα serum levels 12 h p.i.
Herpes simplex virus-2 (HSV-2)	pDCs	i.v. infection, pDC depletion by anti-CD317 (115, 116) or in CLEC4A-DTR-tg mice (65, 114)	↓ Serum IFNα levels 6/8 h p.i. ↑ viral titers in the liver ↓ survival
		Intravaginal infection, pDC depletion by anti-CD317 (117)	↓ Survival ↓ Local IFNα levels
	non-pDCs	Intravaginal infection, pDC depletion in CLEC4A-DTR-tg mice (65, 114)	= Survival = Local IFNα levels
Vaccinia virus (VV)	Inflammatory monocytes	Monocyte depletion in CD11b-DTR-tg mice (71, 104)	↓ Type I IFN serum levels ↑ Viral titers
Modified vaccinia virus Ankara (MVA)	Cells other than pDCs	Footpad infection, pDC depletion in CLEC4A-DTR-tg mice (118)	= Type I IFN levels in draining lymph nodes
Ectromelia virus	Inflammatory monocytes	i.p. infection, monocyte depletion by clodronate liposomes or pDC depletion by anti-BST2 mAb 927; *ex vivo* sorting of inflammatory monocytes and RT-PCR for type I IFN (119)	↓ Type I IFN levels in draining LN after clodronate treatment = Type IFN I serum levels after pDC depletion
Adenovirus and adenoviral vectors	cDCs	pDC depletion by anti PDCA-1 and cDC depletion in CD11c-DTR-tg mice (70, 105)	↓ Type I IFN serum levels after cDC depletion = Type IFN I serum levels after pDC depletion
influenza virus	pDCs	Intranasal infection, pDC-deficient Ikaros^L/L^ mice (120)	= Virus titers = Weight loss as compared to WT; type I IFN levels not assessed
Thogoto virus	CD11b^+^ F4/80^+^ myeloid cells	i.p. infection, FACS analysis and luciferase of peritoneal exudate cells from IFNβ^mob/mob^ and IFN-β^+/Δβ−*luc*^ reporter mice 6 and 18 h p.i. (59, 60, 121)	n.d.
Encephalomyocarditis virus (EMCV-D)	cDCs	cDC depletion in CD11c-DTR-tg mice (70, 106)	↓ Type I IFN serum levels ↑ Viral titers ↑ Diabetes
La Crosse virus, Rabies virus, Theiler's murine encephalomyelitis, vesicular stomatitis virus (VSV)	Astrocytes (to a lesser extent microglia/macrophages and neurons)	histology in IFN-β^+/Δβ−*luc*^ and IFN-β^floxβ−*luc*/*floxβ*−*luc*^ immunostaining and RNA *in situ* hybridization (122, 123)	n.d.
Vesicular stomatitis virus (VSV)	pDCs	i.v. injection, pDCs depletion in CLEC4A-DTR-tg mice (65)	↓ IFNα serum levels ↑ viral titers 6 h p.i. but not at 12 or 24 h p.i.
	Macrophages, pDCs	s.c. infection, pDC depletion by anti-PDCA1 or LN macrophage depletion by clodronate liposomes or in CD11c-DTR-tg mice (70, 108)	↓↓ IFNα (90%) in macrophage depleted LNs ↓ IFNα (50%) in pDC depleted LNs
La Crosse virus	Astrocytes, microglia, neurons	i.p. infection, histology in IFN-β^+/Δβ−*luc*^ and IFN-β^floxβ−*luc*/*floxβ*−*luc*^ immunostaining and RNA *in situ* hybridization (122, 123)	n.d.
Newcastle disease virus (NDV)	pDCs, cDCs, macrophages, monocytes	systemic NDV infection, FACS analyses in Ifna6^gfp/+^ reporter mice (46)	n.d.
	Alveolar macrophages	intranasal NDV infection, FACS analyses in Ifna6^gfp/+^ reporter mice (46)	n.d.
Mouse hepatitis virus (MHV)	pDCs	i.p. infection, pDC depletion anti-CD317 or absence of pDCs in Itgax-Cre^+^ Tcf4^flox/−^ mice (100, 124)	↓ IFNα serum levels ↑ Viral titers increased 48 h p.i.
Lymphocytic choriomeningitis virus (LCMV)	pDCs (and macrophages and cDCs)	WE, i.v. infection, 24–48 h p.i., Ifna6^gfp/+^ reporter mice (76)	n.d.
	Non-pDCs	Armstrong and Clone13, i.v. infection, pDC depletion in CLEC4A-DTR-tg mice (65, 125)	↓ IFNα serum levels 16 h p.i. = At later timepoints
	Non-pDCs	Armstrong and WE, i.v. infection, pDC depletion by anti-Ly6G/C or absence of pDCs in Itgax-Cre^+^ Tcf4^flox/−^ mice (81, 100)	= IFNα serum levels 48h p.i., virus cleared from organs day 8 p.i.
	Non-pDCs	Docile, i.v. infection, high dose, absence of pDCs in Itgax-Cre^+^ Tcf4^flox/−^ mice (100)	Persistent serum virus titers
	Cells other than macrophages	WE, i.v., 48 h p.i., phagocyte depletion by clodronate liposomes, FACS analysis IFNβ^mob/mob^ mice (94)	= IFNα serum levels
	Phagocytic cells	Armstrong, i.v., 48 h p.i., phagocyte depletion by clodronate liposomes (95)	↓↓ IFNα serum levels
	CD169^+^ macrophages	marginal metallophilic macrophage and subcapsular sinus macrophage depletion in CD169-DTR-tg mice (72, 107)	↓ Type I IFN from day 4 p.i. on, persistent virus titers
Chikungunya virus	pDCs	s.c. infection, restriction of type I IFN expression to pDCs in pDC:Irf7^+^ mice (69)	pDC:Irf7^+^ mice protected against infection, 100% lethal in IRF3/7 double deficient mice
Dengue virus	pDCs and cells other than pDCs	i.v. infection, restriction of type I IFN expression to pDCs in pDC:Irf7^+^ mice (69)	↓ Viral titers in pDC:Irf7^+^ as compared to IRF3/7 double deficient mice transiently 42–72 p p.i.
Respiratory syncytial virus (RSV)	pDCs	Intratracheal infection, pDC depletion by anti-CD317 (126)	↑ Viral titers ↑ Immunopathology in the lung day 9 p.i., ø IFNα production in the lung

### DNA Viruses

Findings on the cellular sources of type I IFN during relevant infection models for DNA viruses and the respective *in vivo* experimental strategies are highlighted in the following sections.

#### Human and Mouse Cytomegalovirus

Infection with the human cytomegalovirus (HCMV) causes mostly asymptomatic, latent infections in the immunocompetent host. In immunosuppressed individuals or newborns infected *in utero*, an infection with this virus can lead to severe illness and permanent organ damage. The murine cytomegalovirus (MCMV) exhibits high structural and biological similarity to HCMV and is thus widely used as a model system for antiviral immune responses (128). MCMV induces a biphasic type I IFN response, with peak expressions occurring at 8 h and 36–72 h p.i. which are triggered by the initial virus contact and viral particles entering the system after completion of the first viral replication cycle, respectively (110). Early type I IFN expression is independent of TLR signaling and predominantly generated by stromal cells infected by the virus (110). Using IFNβ^mob/mob^ reporter mice, IFNβ production was detected in splenic pDCs as early as 6–12 h p.i. (59, 111). After *in vivo* depletion of pDCs by anti-CD317 or anti-Ly6G/C treatment IFNα serum levels were severely reduced 36 h after MCMV infection (18, 80, 81, 113). Under these conditions, however, other cell types secrete IL-12 and ensure sufficient IFNγ and NK cell responses leading to control of MCMV infection (18, 80). Of note, 44 h after MCMV infection IFNα serum levels in pDC depleted mice were no longer reduced as compared to untreated mice (113). Similar observations were made in Ik^L/L^ mice that lack pDCs (61) or CLEC4A-DTR-tg mice that have been transiently depleted of pDCs (65, 113). Thus, transient type I IFN production at the first day of MCMV infection was pDC-dependent, while cells other than pDCs are responsible for the type I IFN levels measured at later timepoints, at least when relatively high inocula of MCMV are used. In contrast, at lower doses of MCMV which are presumably closer to a natural infection setting, pDCs can limit viral burden in the spleen and liver. Here, pDCs have been shown to promote NK cell activation and cytotoxicity in the early phase of MCMV infection (65). While it is well-established that pDCs sense the MCMV via the TLR9 and TLR7 mediated pathways (18, 80, 113, 129, 130), also the TLR3 and TLR2 pathways which are functionally used by other cells than pDCs have been shown to be involved in the induction of type I IFN production (104, 131, 132). These findings are in accordance with multiple observations that defects in MyD88 signaling have a more severe impact on anti-MCMV immune responses than TLR9 deficiency or pDC depletion (113, 129). So far, the identity of the non-pDC cell types involved in anti-MCMV type I IFN response remain incompletely defined.

#### Vaccinia Virus

One report indicated that vaccinia virus (VV) and to a lesser extend MCMV induce type I IFN in CD11c^−^ CD11b^+^ Ly6C^+^ inflammatory monocytes, but not macrophages or other types of DCs, in a TLR2 dependent way using IFNβ^mob/mob^ reporter mice. Further, CD11b-DTR-tg mice depleted of monocytes exhibited increased viral titers in the liver and decreased serum levels of type I IFN after VV infection (104). This is similar to other studies using footpad infection of modified vaccinia virus Ankara (MVA) and pDC depletion in the CLEC4A-DTR-tg mouse model, where type I IFN levels in the draining lymph nodes were comparable to control mice indicating that pDCs are not required for mounting an intact type I IFN response after local infection with this dsDNA virus (118).

#### Adenovirus

The dsDNA adenovirus is used as a vector for the development of gene therapy applications but can also cause severe disease in immunocompromised individuals. By using CD11c-DTR-tg mice and anti-CD317 treatment to ablate cDCs vs. pDCs *in vivo* it has been shown that wildtype (WT) adenovirus as well as adenoviral vectors induce rapid IFNα/β production almost exclusively in splenic cDCs rather than in pDCs (105).

#### Herpes Simplex Virus

For Herpes Simplex Virus (HSV) local (subcutaneous or genital) as well as systemic (i.v.) infection models have been analyzed. After subcutaneous HSV-1 infection, pDCs were shown to provide type I IFN necessary for licensing of cDCs which in turn induce effective cytotoxic T cell responses. Here, mice depleted for pDCs by anti-Ly6G/C treatment displayed increased viral titers in the draining lymph nodes at day 7 p.i. as compared to controls (82). Similarly, in a genital HSV-2 herpes model, mice depleted for pDCs using anti-CD317 antibodies succumbed earlier to the infection and exhibited reduced local IFNα levels, while the Th1 response in draining lymph nodes developed normally (117). In contrast to findings from antibody-mediated depletion, in pDC depleted CLEC4A-DTR-tg mice neither differences in viral burden nor survival after vaginal HSV-2 infection was found nor were pDCs found to contribute significantly to antiviral CD8 T cell responses after subcutaneous HSV-1 inoculation (114). These contradicting findings have been explained by the antibody-mediated depletion of additional cell types other than pDCs in contrast to the more restricted depletion in the CLEC4A-DTR-tg genetic mouse model. On the other hand, it cannot be excluded that DTR mediated depletion is less effective and therefore a residual pDC activity retained after DT administration. Slight differences in the respective experimental settings might contribute as well as e.g. after antibody-mediated pDC depletion IFNα levels were measured in vaginal washes while in the genetic depletion model total protein amount was assessed in the vaginal and cervical tissue itself. As for MCMV, TLR3-expressing cells, such as CD8^+^ DCs or other hematopoietic and non-hematopoietic cells, are essential for type I IFN production in local HSV infection at later timepoints rather than pDCs (114).

After systemic challenge with UV-irradiated HSV in an early study immunohistological stainings for IFNα/β indicated that the majority of type I IFN producing cells in the spleen represent marginal metallophilic macrophages and to a lesser extend MZMs (133). However, IFNα levels were markedly reduced in pDC depleted Siglech^dtr/dtr^ mice 6 h after i.v. infection with HSV-1 and viral titers were found increased in the spleen as compared to control animals pointing toward pDCs as the major type I IFN producers in this situation (66). Similar results were obtained in pDC depleted CLEC4A-DTR-tg mice, with the exception that no viral replication was detectable in the spleens of either DT-treated CLEC4A-DTR-tg or control mice (114). This discrepancy may reflect differences in the strains of HSV-1 used or differences in the promoters used to drive DTR expression (CLEC4A vs. SiglecH) with a slightly divergent expression pattern as discussed above. For systemic HSV-2 infections, results from antibody depletion and pDC ablation in CLEC4A-DTR-tg mice corresponded well since in both cases a reduction of IFNα serum levels were observed together with increased viral titers and reduced survival (114–116). Thus, similar to vaccinia virus the cell type responsible for the production of type I IFN in HSV infection may depend on the route of pathogen entry with pDCs controlling the infection once the virus has spread systemically.

#### Ectromelia Virus

Ectromelia virus (ECTV), a large DNA orthopoxvirus, is the causative agent of mousepox, the mouse homolog of human smallpox. ECTV causes systemic disease after s.c. infection of the footpad. *In vivo* it was shown by clodronate and anti-CD317 mediated depletion of monocytes vs. pDCs and *ex vivo* sorting and RT-PCR analyses that infected inflammatory monocytes are the major producers of type I IFN in the draining lymph nodes (119).

In summary, the cellular source for type I IFN production during DNA virus infection depends on the virus type itself, the dosage, timepoint as well as route of infection. Early after infection with MCMV pDCs are the primary source of type I IFN production capable of reducing virus titers at low concentrations of the virus. However, at later timepoints of infection CD8^+^ DCs rather than pDCs become the key source of type I IFN production. In addition to pDCs, other cell types such as cDCs in adenovirus infection, metallophilic macrophages and MZMs during HSV exposure, stromal cells in MCMV infection and inflammatory monocytes in response to ECTV are an essential source of type I IFN.

### RNA Viruses

A recent meta-transcriptomics survey defined 196 vertebrate-specific RNA virus species the majority of which is able to infect humans and cause diseases of varying severity (134, 135). At the moment only few mouse models are available to elucidate the host immune response to these viruses. In this chapter we summarize the *in vivo* model studies aimed at visualizing type I IFN producing cell types and defining their contribution to the type I IFN production and RNA virus control.

#### Newcastle Disease Virus

For systemic infections with RNA viruses, such as after i.v. inoculation with the paramyxovirus Newcastle disease virus (NDV), it has been shown that pDCs and also cDCs, macrophages, and monocytes, produced IFNα (46). Here, pDCs mount an antiviral type I IFN response in a viral replication-independent manner through virus recognition by TLR7 and the activation of the type I IFN positive feedback loop. Only in the absence of this type I IFN positive feedback, the virus infects and also replicates in pDCs. In this case, type I IFN induction occurs in pDCs via cytoplasmic RLHs (32). However, other ssRNA viruses have been reported to induce type I IFN expression in pDCs in a replication dependent manner (136, 137). Especially for vesicular stomatitis virus (VSV), the capture of the replicating virus in the autophagosome is required for its transfer to the TLR7 containing endosomal compartment (137). After local infection with NDV, here after intranasal infection, the IFNα-producing cells shifted from pDCs to alveolar macrophages and cDCs that utilize the RLH system for type I IFN induction (46).

#### Vesicular Stomatitis Virus

Upon s.c. VSV infection, draining LNs contained ~90% less IFNα when depleted of macrophages by clodronate liposomes. However, when pDCs were depleted by anti-CD317 treatment, IFNα levels induced by VSV were reduced only by half as compared to controls. It was concluded that infected CD169^+^ subcapsular sinus macrophages produce IFNα, yet half of the type I IFN is produced by pDCs stimulated directly or indirectly by the infected macrophages (108). Later it was shown that CD169^+^ macrophages in the spleen represent a compartment of enhanced viral replication (138). Thus, it is conceivable that CD169^+^ macrophages potentiate the type I IFN response indeed indirectly via activating pDCs. When VSV was inoculated i.v. in CLEC4A-DTR-tg mice transiently depleted for pDCs, IFNα was found reduced and viral titers increased only at very early timepoints, again pointing to a rather transient role of pDCs in anti-viral immunity (65).

#### Dengue and Chikungunya Virus

For the distantly related arboviruses Dengue (DENV) and Chikungunya (CHIKV) virus it was recently shown that pDCs are sufficient to control these viruses via IRF7-regulated type I IFN responses in both systemic as well as local infection settings. In this report novel pDC:Irf7^+^ mice were introduced in which IRF7-driven type I IFN production is restricted to pDCs and were compared to IRF3/7 double deficient mice that are completely devoid of type I IFN expression (69). After i.v. infection with DENV pDC:Irf7^+^ mice exhibited a lower viral load than Irf3/7 double deficient mice. However, as compared to WT mice higher viral tiers were detected in pDC:Irf7^+^ mice (69). After s.c. infection with CHIKV Irf3/7 double deficient mice succumb to the virus while 100% of pDC:Irf7^+^ mice survive the infection exhibiting no overt clinical symptoms similar to WT mice. Early control of viremia in pDC:Irf7^+^ mice was reduced as compared to WT but still improved as compared to Irf3/7 double deficient mice. Thus, analogous to findings from other virus infection models also for these RNA viruses, antiviral response mounted by pDCs controls infection once the virus spreads systemically.

#### La Crosse Virus, Rabies Virus, and Theiler's Murine Encephalomyelitis Virus

In infection models with RNA viruses exhibiting a specific tropism, pDCs play only a minor role. In the brain of mice infected with the ssRNA La Crosse virus, IFNβ production was assessed by the IFN-β^+/Δβ−luc^ luciferase reporter mouse model (60) and detected in astrocytes, microglia, and to a lesser extend also in infected neurons (122). This confirmed earlier findings where IFNα/β expression in these cell types after La Crosse virus infection was visualized by immunostaining and RNA *in situ* hybridization (123). Utilizing the conditional reporter activity of the IFN-β^floxβ−luc/floxβ−luc^ mice it was shown for several neurotropic viruses such as rabies virus (RABV), Theiler's murine encephalomyelitis virus (TMEV), and VSV that astrocytes are the main producers of IFNβ after infection of the brain (139).

#### Encephalomyocarditis Virus

Another example for type I IFN expression by non-myeloid cells represent β-islet cells. The encephalomyocarditis virus (EMCV) strain D, an ssRNA picornavirus with tropism for the insulin-producing β cells of the pancreas, can induce diabetes and myocarditis in certain mouse strains. CD11c^+^ cells in this model have been shown to be protective as DT treated CD11c-DTR-tg mice developed diabetes and exhibited increased viral titers in the pancreas, spleen, and heart associated with reduced type I IFN levels as compared to non-depleted controls (106).

#### Pneumonia Virus of Mice

Pneumonia virus of mice (PVM) infection led to a marked infiltration of pDCs and increased expression of type I IFN in WT but not TLR7- or MyD88-deficient mice. Transfer of TLR7-competent, but not TLR7-deficient pDCs led to a significantly diminished virus recovery in TLR7^−/−^ animals on day 7 after infection with PVM indicating that TLR7-mediated signaling by pDC is required for appropriate innate responses to acute PVM infection (140).

#### Respiratory Syncytial Virus

For intratracheal infection with respiratory syncytial virus (RSV) it has been shown that anti-CD317 mediated depletion of pDCs completely abolished IFNα expression and protein levels in the lungs. This correlated with increased viral titers and exacerbated immunopathology of the lungs of pDC depleted mice (126). Thus, pDCs fulfill a substantial protective role during local RSV infection.

#### Influenza Virus and Influenza Virus-Like Orthomyxovirus Thogoto Virus

Initial *in vitro* studies showed that spleen cells from mice that were depleted for pDCs by anti-Ly6G/C injection did not produce IFNα in response to stimulation with inactivated influenza virus in contrast to splenocytes from untreated animals (18). IFNα production *in vitro* could be attributed to the CD317^+^ CD11c^+^ pDC population of sorted mouse spleen cells (86). However, *in vivo* intranasal infection with sublethal doses of influenza virus in pDC-deficient Ikaros^L/L^ and WT mice revealed a similar course of disease, as determined e.g. by weight loss and viral titers (120). Thus, pDCs are able to produce type I IFN after stimulation by influenza but are dispensable for a successful antiviral immune response. Albeit, type I IFN levels *in vivo* were not assessed for this infection model.

For the influenza virus-like orthomyxovirus Thogoto virus (THOV) type I IFN production in the peritoneal cavity was mainly attributed to CD11b^+^ F4/80^+^ myeloid cells that was independent of the type I IFN receptor mediated feedback loop and coincided with the tropism of this virus (121).

#### Mouse Hepatitis Virus

After i.p. infection with Mouse hepatitis virus (MHV), pDC depletion by anti-CD317 was accompanied by severely diminished IFNα serum levels (124). The transient pDC depletion did not lead to lethality following the low-dose MHV infection used in this study. Nevertheless, initial viral titers in spleens were found increased more than 1,000-fold in pDC-depleted compared to control mice (124). Very similar observations were made in Itgax-Cre^+^ Tcf4^flox/−^ mice lacking pDCs. These mice show reduced serum IFNα levels and elevated viral loads in the liver and spleen (100). Thus, pDCs appear to be essential for type IFN I mediated protection against systemic infection with the prototypical acute cytopathic coronavirus MHV.

#### Lymphocytic Choriomeningitis Virus

Lymphocytic choriomeningitis virus (LCMV) infection is widely used to study acute as well as chronic infections. In an acute infection setting in Ifna6^gfp/+^ reporter mice pDCs were found to be the major type I IFN producers after infection with the WE strain of LCMV. Additionally, few cDCs and macrophages specifically in the spleen exhibited GFP-reporter activity (76). Also, in IFNβ^mob/mob^ reporter mice macrophages could be excluded as major type I IFN producers and depletion of phagocytic cells by clodronate liposomes did not affect type I IFN serum levels (94). In contrast, another study using the Armstrong strain of LCMV reported severely reduced IFNα/β serum levels after clodronate treatment (95). Specifically, a small population of CD169^+^ macrophages in the spleen and lymph nodes has recently been shown to release high amounts of type I IFN after LCMV infection. Selective depletion of these cells in CD169-DTR-tg mice resulted in reduced type I IFN levels from day 4 p.i. onward and persistent viral titers. As a consequence, CD169 depleted mice exhibited severe immunopathology and died quickly after infection (107). In line with this, production of serum type I IFN was not reduced in LCMV infected mice treated with the pDC depleting anti-Ly6G/C antibody as compared to those injected with control antibody (81). Also, in congenitally pDC-deficient Itgax-Cre^+^ Tcf4^flox/−^ mice, virus titers early after infection were comparable to WT controls confirming that pDCs are dispensable for the control of acute LCMV infection (100). Still, pDCs have been shown to be a transient source of type I IFN as pDC depletion in CLEC4A-DTR-tg mice led to reduced serum IFNα levels at 16 h p.i. with LCMV Armstrong or clone 13, but not at later timepoints (125). Contrasting the observations in acute LCMV infection, in a chronic infection setup using LCMV Docile the virus persisted until day 53 in the blood of Itgax-Cre^+^ Tcf4^flox/−^ mice while the virus was cleared between day 21 and 28 in WT mice. This was attributed to a failure of sufficient CD4^+^ and CD8^+^ T cell activation in the absence of pDCs and indicated that pDCs are essential for generating a functional adaptive immunity to chronic viral infections (100).

Taken together, pDCs are a major source of type I IFN and are required for type I IFN mediated protection against systemic infection in most of the RNA virus infections such as NDV, VSV, DENV, CHIKV, PVM, RSV, MHV, and LCMV. However, contribution of pDCs in type I IFN release and type I IFN mediated protection depends on the titer of the virus, time after infection, and the route of the infection. In addition to pDCs, other cell types such as cDCs, macrophages, and monocytes in NDV, macrophages in VSV, astrocytes, microglia, and neurons in La Crosse virus, astrocytes in RABV, TMEV, and VSV, β-islet cells and cDCs in EMCV, and cDCs and macrophages in LCMV infection significantly contribute to type I IFN production. Thus, similar to infection with DNA viruses, also after infection with RNA viruses pDCs are functionally involved in type I IFN production mostly early during infection but are dispensable for virus control during later stages of infection. In chronic infection, however, pDCs provide type I IFN to support and preserve T cell functions.

### Retroviruses

HIV activates pDCs to produce high levels of IFNα most likely via activation of TLR7 (141). Also, it is assumed that type I IFNs are produced during HIV infection predominantly by pDCs as decreased IFNα production in HIV-infected patients correlates with numerical and functional deficiencies in circulating pDCs (142). A direct assessment of the contribution of pDCs to type I IFN levels in HIV-infection, however, has not been performed. Although type I IFNs are known to mediate antiviral immunity, there has always been caution toward a detrimental role of type I IFNs during HIV/AIDS because of their proinflammatory nature (11, 143, 144). Thus, many studies have shown that pDCs are a source of type I IFN in retroviral and other virus infections *in vivo*. However, additional cellular sources of type I IFN are required to fully control viral infections. In summary, pDCs are a known source of type I IFN in retroviral infection. However, the relative contribution of pDCs vs. other type I IFN producers to the overall type I IFN response and immune control or pathology after retrovirus infection, is not fully understood.

## Bacterial Infections

While a considerable number of studies have been undertaken to define the cellular source of type I IFN and the functions of these cell types in viral infections, fewer data exist for non-viral infections. In bacterial infections, type I IFNs can act as activators of protective immune responses or mediate immunosuppressive functions leading to exacerbation of the infection. This ambivalent role of type I IFN has been reviewed recently (1, 11, 145). In this chapter we will focus on the efforts to clarify the identity and impact of type I IFN producing cells as knowledge on these has increased significantly paralleling the availability of newly developed mouse models.

### Mycobacteria

It has been well established that CD4 T cells as well as secreted effector cytokines TNF, IL-12, and IFNγ exert protective functions in host resistance to the intracellular bacterial pathogen *Mycobacterium tuberculosis* (*Mtb*) (146). In contrast, the role of type I IFN during *Mtb* infection appears to promote infection instead of controlling infection. Type I IFNs downregulate IFNGR1 expression and thereby suppress IFNγ signaling (147, 148) and IFNAR-deficient mice displayed increased bacterial clearance to infection with *Mtb*, although bacterial growth in the lung was unaffected (149). In *in vitro* studies, BM-derived macrophages and DCs have been identified as a possible source of type I IFN in response to *Mtb* (149, 150). Also, human peripheral blood mononuclear cell (PBMC)-derived macrophages and especially DCs were shown to produce type I IFN after *in vitro* infection with *Mtb* (151, 152).

Similar to *Mtb*, IFNγ promotes antimicrobial activity against *Mycobacterium leprae* whereas type I IFNs contribute to pathogenesis (153). Here, PBMC-derived monocytes expressed IFNβ and IFN-stimulated genes including the immunosuppressive cytokine IL-10 during *M. leprae* infection *in vitro* (153). So far the cell type expressing type I IFN in the context of mycobacterial infections *in vivo* as well as definition of the functional impact of these cells await clarification.

### Listeria monocytogenes

Type I IFN not only inhibits antibacterial signaling pathways and promotes infection in the case of mycobacteria. Also, *L. monocytogenes* has evolved mechanisms to activate the type I IFN pathway for the benefit of this intracellular pathogen. Mice deficient in IFNAR signaling are more resistant to systemic *L. monocytogenes* infection as compared to WT controls. Mechanistically, type I IFNs enhance susceptibility to systemic *Listeria* infection by reducing responsiveness to IFNγ, decreasing the number of pro-inflammatory myeloid cells, promoting the expression of proapoptotic genes, and enhancing T cell sensitivity to apoptosis (148, 154–156). Of note, in intragastric or foodborne infection with *L. monocytogenes* type I IFN receptor mediated signaling contributed positively to survival of infected mice or did not have an impact at all, respectively (157, 158). This emphasizes again, that the route of infection contributes significantly to differences in the impact of type I IFN in infection.

Four distinct cell types have been reported as sources for type I IFN production during systemic *L. monocytogenes* infection *in vivo* (Table 3). For one, a FACS-purified splenic cell population from infected mice that displays surface antigens typical of macrophages and not pDCs was identified as the main producer of type I IFN (159). Also, the apathogenic *Listeria* mutant lacking listeriolysin O which is unable to escape from the phagolysosome into the cytoplasm of the infected cell, does not stimulate IFNβ synthesis (164, 165). Later, Tip-DCs, an effector subtype of Mac-3^hi^ inflammatory monocytes, which produce TNF and iNOS were identified as the major IFNβ-producing cells *in vivo* in systemic *L. monocytogenes* infections using IFNβ^mob/mob^ and IFN-β^+/Δβ−*luc*^ reporter mice (160, 161). IFNβ-producing TiP-DCs harbored high bacterial loads and were located within the foci of infection in the splenic white pulp ideally positioned to activate T cells as well as NK cells via type I IFN (160). Bacterial loads in the spleen were severely increased in mice deficient in CCR2 and thus lacking TiP-DCs (162). Thus, this subtype of inflammatory monocytes has been attributed an important role in early containment of *L. monocytogenes* infection (162, 166). The overall role of TiP-DCs in this infection may therefore be ambiguous, having a regulatory function in controlling the balance between containment of infection and at the same time mediating detrimental effects of type I IFN on the host. Interestingly, in the spleens of *Listeria*-infected CCR2^−/−^ mice increased levels of type I IFN were observed indicating that alternative cell types produce type I IFN in the absence of TiP-DCs which are triggered additionally by increased bacterial load. Along this line, a detrimental role for pDCs in controlling *L. monocytogenes* infection was demonstrated in *Siglech*^dtr/dtr^ mice where ablation of pDCs caused significantly increased survival and decreased bacterial burden at day 3 p.i., while type I IFN levels themselves were not analyzed under these conditions (66). At earlier timepoints, however, anti-PDCA-1 mediated depletion of pDCs did not lead to a difference in bacterial load or levels of type I IFN in the spleen as compared to control animals (161). In one report, CD317^+^ SiglecH^−^ CD19^+^ B cells have been found to be able to induce IFNα after stimulation with heat-killed *L. monocytogenes* (163). *Ex vivo* isolated CD317^+^ SiglecH^−^ CD19^+^ B cells activated cytotoxic function of NK cells in an IFNα-dependent manner. *In vivo*, this B cell subset contributed positively to resistance to *L. monocytogenes* infection as Btk^−/−^ mice deficient for B-cells and unable to generate CD317^+^ CD19^+^ B cells displayed increased susceptibility to *L. monocytogenes* infection, while adoptive transfer of CD317^+^ CD19^+^ B cells to Btk^−/−^ mice normalized their resistance to *L. monocytogenes* infection (163).

**Table 3:** Cellular sources of type I IFN production in *Listeria monocytogenes* infection *in vivo*.

**Type I IFN producing cells**	**Model systems and assay methods**	**Observations in the absence of cell type**
Macrophages	i.p. infection, RT-PCR for type I IFN from *ex vivo* FACS sorted cell populations (159)	n.d.
Tip-DCs	i.v. and i.p. infection, FACS analysis and histology in IFNβ^mob/mob^ and luciferase activity in IFN-β^+/Δβ−*luc*^ reporter mice (59, 60, 160, 161)	n.d.
Non-TiP-DCs	i.v. infection, TiP-DC deficient CCR2^−/−^ mice (162)	↑ Bacterial load ↑ Type I IFN in the spleen
pDCs	i.p. infection, pDC depletion in S*iglech*^dtr/dtr^ mice (66)	↑ Survival ↓ Proinflammatory cytokines
PDCA-1^+^ SiglecH^−^ CD19^+^ B cells	i.p. infection, B cell deficient Btk^−/−^ mice (163)	↓ Survival

### Extracellular Bacteria

As for intracellular bacteria also for extracellularly replicating bacterial pathogens type I IFN can either be detrimental or essential for host defense (145). Group B streptococci (GBS) are important neonatal pathogens and type I IFN receptor signaling is reported to contribute to host resistance against this pathogen (167). Mice i.p. infected with GBS express elevated levels of IFNβ and IFNα4 mRNA in the spleen. *In vitro*, GBS activated type I IFN expression in peritoneal macrophages, BM-derived cDCs and to a lesser extent also in macrophages, while pDCs were completely unable to produce type I IFN after GBS stimulation (167, 168).

In contrast to GBS, type I IFN induction in the mixed bacterial sepsis model of colon ascendens stent peritonitis (CASP) has been shown to have a detrimental effect on the host. Septic peritonitis induced in IFNAR^−/−^ mice showed improved survival and bacterial clearance as compared to WT controls. Splenic CD11b^+^ CD11c^−^ macrophage-like cells could be identified as major producers of IFNβ *ex vivo* by RT-PCR analyses from sorted cells, while no IFNα subtypes were detected (169).

In summary, type I IFN production has a detrimental effect for the host after infection with intracellular bacteria such as mycobacteria and *L. monocytogenes*. BMDCs and PBMC-derived DCs and macrophages are the responsible cell types for type I IFN production during mycobacteria infection. For *L. monocytogenes*, four cell types have been identified as type I IFN producers, namely macrophages, Tip-DCs, inflammatory monocytes, and B cells. In the case of extracellular bacteria, the cell types identified as type I IFN producers include macrophages and BMDCs. However, with the exception of the intracellular model organism *L. monocytogenes*, the knowledge on the cellular source of type I IFN in bacterial infection is rather scarce.

## Fungal Infections

As for bacterial infections, the effect of type I IFN in mouse models for infections with pathogenic fungi has been reported as beneficial or detrimental for the host depending on the fungal species and the route of infection. Additionally, controversial results obtained from very similar infection settings have been explained by the possible impact of differences in the microbiome in the respective mouse colonies (1, 11). The cell type responsible for type I IFN production in fungal infections *in vivo*, however, awaits clarification. To our knowledge only for the important opportunistic fungal pathogen *Aspergillus fumigatus in vivo* studies in this direction have been undertaken. The type I IFN response triggered by *A. fumigatus* was analyzed initially in human pDCs isolated from PBMCs. When these cells were stimulated *in vitro* with *A. fumigatus* hyphae IFNα was detected in the supernatant (170). IFNAR^−/−^ mice or mice depleted of pDCs by anti-CD317 treatment exhibited an increased susceptibility to pulmonary or i.v. infection with *A. fumigatus* conidia. A direct impact of pDC depletion on type I IFN levels *in vivo* after infection, however, has not been analyzed in this study (170). Therefore, the hypothesis that pDCs mediate their protective function in this fungal infection directly via type I IFN remains to be tested.

## Infections With Protozoan Parasites

Infection with a wide variety of protozoan parasites can trigger type I IFN expression in mammalian hosts as reviewed recently (171, 172). For *Plasmodium, Leishmania*, and *Trypanosoma in vivo* infection models several studies have been carried out in the last few years which allowed the identification of cellular sources of type I IFN in response to intracellular parasite infections. This will be the focus of the following chapter and summarized in Table 4.

**Table 4:** Cellular sources of type I IFN production in intracellular parasite infections *in vivo*.

**Parasite**	**Type I IFN producing cells**	**Model systems and assay methods**	**Observations in the absence of cell type**
*Plasmodium berghei* ANKA	pDCs and (CD8^−^) cDCs	RT-PCR from *ex vivo* purified splenic pDCs and cDCs (173, 174)	n.d.
	cDCs	Phagocyte depletion by clodronate liposomes, cDC specific IFNAR deficiency in CD11c-Cre Ifnar1^fl/fl^ mice	↓ IFNα serum levels
*Plasmodium chabaudi*	pDCs	RT-PCR for type I IFN from *ex vivo* purified splenic pDCs; pDC depletion by anti-CD317 (175)	= Parasite clearance
	pDCs and red pulp macrophages (RPMs)	FACS analysis in IFNβ^mob/mob^ reporter mice, RT-PCR from *ex vivo* purified splenic cell populations, pDCs; pDC depletion by anti-CD317, RPM deficient SpiC^−/−^ mice (176)	↓ IFNα levels in the spleen = Parasite clearance
*Plasmodium yoelii YM*	pDCs	FACS analysis in IFNβ^mob/mob^ reporter mice (59, 177), pDC depletion by anti-CD317 (178) or CLEC4A-DTR-tg mice (65, 177)	↓ IFNα serum levels, ↑ Parasitemia
*Leishmania donovani*	B cells	RT-PCR for type I IFN from *ex vivo* purified B cells (179)	n.d.
*Toxoplasma gondii*	Intestinal epithelial or lamina propria cells	RT-PCR for type I IFN from *ex vivo* purified intestinal epithelial or lamina propria cells (180)	n.d.
	Inflammatory monocytes	RT-PCR for type I IFN from *ex vivo* depleted cell populations (181)	n.d.

### Plasmodium

Malaria is an important parasitic disease predominantly in tropical and subtropical African regions. It is caused by the protozoan parasite *Plasmodium* with *P. falciparum* being responsible for its most severe forms. In humans, malarial parasites are transmitted at sporozoite-stage by infected mosquitoes (182). The transmitted sporozoites rapidly travel to the liver, where they infect hepatocytes and initiate clinically silent but immunologically active liver-stage infection (171). Well-established *in vivo* mouse models include the lethal *Plasmodium yoelii* YM and *P. berghei* ANKA leading to high parasitemia and cerebral malaria (CM), respectively, after inoculation with *Plasmodium*-infected erythrocytes. Further, *P*. *chabaudi* is used as a chronic infection model. Various cellular sources for type I IFNs have been proposed after *Plasmodium* infection *in vitro* (182–184).

After inoculation with *P. berghei* ANKA infected erythrocytes *in vivo*, isolated splenic pDCs as well as CD8^−^ cDCs expressed type I IFN (173, 174). Using anti-CD317 mediated pDC depletion and cDC depletion in CD11c-DTR-tg mice it was shown that cDCs but not pDCs are required for the induction of CM (173). Additionally, cDCs require IFNAR dependent signaling for systemic IFNα production in this model as indicated by substantially lower levels of serum IFNα in CD11c-Cre Ifnar1^fl/fl^ mice, compared to those in infected *Ifnar1*^*fl*/*fl*^ littermate controls (174).

In contrast to the *P. berghei* ANKA model, *P. chabaudi* infection did not induce IFNα in splenic cDCs but rather in pDCs via the TLR9 sensing pathway (175). However, pDCs were not essential for parasite clearance in *P. chabaudi* infection (175). Direct *in vivo* analysis performed in IFNβ^mob/mob^ reporter mice (59) revealed that in *P. chabaudi* infection about 75% of IFN producing cells are pDCs (176). In addition to pDCs, splenic red pulp macrophages (RPMs) can generate significant quantities of IFNβ in response to *P. chabaudi* infection. Contribution of both cell types to the type I IFN response in this system was defined by pDC depletion via anti-CD317 treatment and in RPM deficient SpiC^−/−^ mice (176).

In the lethal malaria mouse model of *P. yoelii YM* infection, type I IFN enhances inflammatory blood leukocyte activation and lethal outcome (177). IFNβ^mob/mob^ reporter mice indicated here that type I IFN is produced in high amounts by BM and blood pDCs and to lesser extent by tissue resident pDCs (177). Depletion of pDCs by anti-CD317 or using pDC specific CLEC4A-DTR-tg mice confirmed pDCs as the major cellular source of type I IFN in this severe malaria model (177, 178). However, depletion of pDCs also resulted in a slight but significant increase of parasitemia (178). Further, priming of pDCs by plasmodium activated CD169^+^ macrophages was essential (177). It was proposed that in *in vivo* settings the low levels of secreted type I IFN produced by monocytes and macrophages prime pDCs for systemic production of type I IFN in malaria.

From data available so far, pDCs as well as cDCs and macrophage subtypes are the cell types responsible for the generation of the type I IFN response, depending on the *Plasmodium* species. Similar to LCMV, *Mycobacteria*, or *Listeria* infections (153, 156, 185), it is thought that early robust production of type I IFN in the first 24 h is essential to induce protective innate and adaptive immunity against *Plasmodium*, while late production of type I IFN impairs host anti-malaria immune responses by induction of negative immune regulators such as PD-L1 and IL-10 (178).

### Leishmania

*Leishmania* spp. are transmitted to mammalian organisms by the bite of infected sand flies (186). The parasites preferentially infect macrophages, but can also be found in other cell types, such as fibroblasts, neutrophils, and DCs (172). Depending on the parasite species and strain *Leishmania* causes a mild to severe cutaneous, mucocutaneous or visceral leishmaniasis (171, 187). Increased production of type I IFN has been observed in local tissues and in the draining lymph nodes of *L. major* infected mice (187, 188). There are diverging reports about the role of type I IFN production in the control of parasite burden and development of disease pathology. Depending on the time course of infection and type I IFN induction it can exert detrimental or protective effects for the host in Leishmaniasis (189). Most of the studies addressing the cellular source of type I IFN in *Leishmania* infection were performed *in vitro*. For example, infection of murine macrophages with *L. major* or *L. amazonensis* lead to type I IFN production (188, 190). *In vitro* exposure of BM-derived as well as splenic pDCs to *L. major, L. infantum*, or *L. braziliensis* promastigotes induces release of IFNα and IFNβ in a TLR9-dependent manner (191). Intriguingly, the amounts of type I IFN produced in response to *Leishmania* spp. are comparable to the type I IFN levels produced in response to stimulation with CpG ODNs in these experiments (191). Recently *in vitro* exposure to the parasite *L. donovani* was reported to trigger IFNβ production in splenic B cells. Here, also high levels of type I IFN mRNA were detected in splenic B cells purified from *in vivo L. donovani* infected mice (179). Taken together, depending on the *Leishmania* subtypes, pDCs and B cells are the source of type I IFN when the cells are directly exposed to the pathogen *in vitro*. Information on the type I IFN producers *in vivo* remain scarce so far for this important protozoan parasite model but could be increased significantly making use of the now available mouse models.

### Toxoplasma

*Toxoplasma gondii* is an intracellular protozoan parasite that has infected at least 50% of the human population. It causes severe toxoplasmosis in immune-suppressed patients. *T. gondii* can infect a wide range of warm-blooded animals, is able to invade any nucleated cell but survives outside of the mammalian host as well (171, 172). The gut epithelium is a strategic barrier to prevent or limit parasite dissemination upon oral infection with *T. gondii*. In the initial phase of oral *T. gondii* infection elevated IFNβ mRNA levels were observed in the small intestine. Intestinal epithelial cells (IECs) and cells from the lamina propria are the source of local IFNβ production in early infection as assessed by real-time PCR performed on cells isolated from infected mice (180). In *in vitro* infection, *T. gondii* has been reported to induce or suppress type I IFN induction depending on the host species, the cell type, and the parasite strain analyzed. One publication showed that BM-derived murine pDCs produce IFNα after infection with *T. gondii* (192). Murine pDCs recognized *T. gondii* profilin via TLR11 and TLR12 and produce type I IFN in a MyD88 dependent fashion (192, 193). In contrast to murine pDCs, human pDCs lack TLR11 and TLR12 and are unable to produce type I IFN despite of direct infection with *T. gondii*. Active infection with *T. gondii in vitro* rather functionally inactivates human pDCs (194). In particular macrophages and DCs serve as reservoirs of *T. gondii* infection and facilitate early dissemination (195). Most of the *Toxoplasma* strains tested are unable to induce type I IFN production in murine BM-derived macrophages after *in vitro* infection (196, 197). *T. gondii* mediated suppression of type I IFN expression has been reported also for monocytes, macrophages, and several DC subsets *in vitro* (181, 195, 196). On the other hand, few atypical *Toxoplasma* strains such as COUGAR and RUB can induce IFNβ production in murine BM-derived macrophages as well as in human skin fibroblasts in *in vitro* infection systems (196). In a physiological oral infection mouse model *ex vivo* isolated inflammatory monocytes in the gut-draining mesenteric lymph nodes were identified the major producers of IFNβ. The expression of IFNβ by inflammatory monocytes required phagocytic uptake of *T. gondii*, while active invasion did not trigger IFNβ induction (181). Thus, depending on the host species, the cell type, and the parasite strain, *T. gondii* may induce or suppress type I IFN production. Epithelial, skin fibroblasts, pDCs, macrophages and inflammatory monocytes here are known cellular sources of type I IFN. In *T. gondii* infection most of the knowledge about the cellular sources of type I IFN is deduced from *in vitro* experiments. Analysis of type I IFN reporter mouse models with and without ablation of different cell types is missing, so far.

Taken together, the major type I IFN producing cell types and their contribution to immunity against many protozoan parasites remain to be defined. To our knowledge, no direct study to elucidate the cellular sources of type I IFN in multicellular parasite such as helminth infections *in vivo* has been published. In order to understand the cellular sources of type I IFN and their relevance with regard to disease elimination in multicellular parasites such as helminths, type I IFN reporter mouse models and cell specific depletion models remain to be analyzed. As for the other pathogen types reviewed above, parasite numbers and the site of infection might influence the sensing pathway and cell type activated to produce type I IFN.

## Concluding Remarks and Future Perspectives

In recent years the generation of novel animal models has remarkably advanced our understanding of the mode of action of IFNs and the cell type responsible for its production in the context of an infection. The existing knowledge does not allow to depict any cell type as a single cell type responsible for the entire type I IFN production in the course of any infection. Rather, depending on the type of infection a wide variety of cells have exhibited the capacity to produce type I IFN. Decisive factors for the type of cell initiating type I IFN production are the type and amount of pathogen and the site and stage of the infection. Additionally, the genetic background of the mouse model and its microbiome status contribute as well and need to be further analyzed.

Even though pDCs are more specialized than other cell types in type I IFN production, it is getting increasingly clear that *in vivo* their contribution to antiviral immunity and also to immune responses to bacterial, fungal, and parasitic infection exhibits restricted patterns in time of induction and duration. The importance of pDCs as the source of type I IFN early in virus infections does not hold true at later timepoints when other host cells take over as dominant producers of type I IFNs. The impact of pDCs also depends on the route of infection. While pDCs provide an important source of type I IFN in systemic infections, their requirement for I IFN-mediated antiviral immune responses in local tissues seems to be necessary only if other lines of defense are broken. However, there are exceptions to the rule as shown for local infections with MHV and HSV-2 where pDC-derived type I IFN in mice is critical for viral control and survival. Indeed, the limitation of pDC responses is caused by an upregulation of pro-apoptotic molecules and apoptosis induction in pDCs in a type I IFN-dependent manner during systemic viral infections (198). This has been suggested as a mechanism to prevent immunopathology due to sustained pDC-mediated type I IFN production. Besides pDCs, mainly macrophages, inflammatory monocytes and cDCs are able to mount significant anti-infectious type I IFN responses *in vivo*. Instead of a single specialized cell type, it is rather the orchestrated type I IFN expression by multiple cellular sources that ensures protective anti-infectious immune responses mediated by type I IFN. To elucidate synergisms and redundancies between the different type I IFN producing cells will be a topic of future studies.

Advanced single cell functional profiling and systems biology approaches will contribute significantly in the near future to identify the exact functions of specific cell types, even cell subtypes, in the different stages of an infection. The spatio-temporal interaction of the type I IFN producing cell with the pathogen and the immune cells that are activated by type I IFN could help to better dissect the diverse functions of type I IFN in the immune response at different stages of infection. Importantly, due to the severe side effects of type I IFN treatment, there is a dire need to better control its activity and thereby increase its beneficial net effect. Strategies such as modifying the affinity of type I IFNs or modulating its time of availability have been reviewed recently (199). These new approaches to develop and improve vaccination strategies and to define novel therapeutic leads for infectious diseases are urgently called for in a time where antibiotic resistances are projected to increase rapidly.

## Author Contributions

SA, RM-N, AS, LR, JA, and SS wrote the manuscript. SA, RM-N, and SS designed and generated tables. RM-N designed and generated the figure. All authors read and approved the final manuscript.

### Conflict of Interest Statement

The authors declare that the research was conducted in the absence of any commercial or financial relationships that could be construed as a potential conflict of interest.
